# Molecular Determinants for RNA Release into Extracellular Vesicles

**DOI:** 10.3390/cells10102674

**Published:** 2021-10-06

**Authors:** Marie-Luise Mosbach, Christina Pfafenrot, Elke Pogge von Strandmann, Albrecht Bindereif, Christian Preußer

**Affiliations:** 1 Institute of Biochemistry, Justus Liebig University of Gießen, 35392 Gießen, Germany; marie.mosbach@chemie.bio.uni-giessen.de (M.-L.M.); christina.pfafenrot@chemie.bio.uni-giessen.de (C.P.); 2 Institute for Tumor Immunology, Center for Tumor Biology and Immunology (ZTI), Philipps University of Marburg, 35043 Marburg, Germany; poggevon@staff.uni-marburg.de

**Keywords:** extracellular vesicles, exRNA, absolute RNA quantification, Y1 RNA, U1 snRNA, U6 snRNA, GAPDH mRNA

## Abstract

Extracellular vesicles (EVs) are important for intercellular communication and act as vehicles for biological material, such as various classes of coding and non-coding RNAs, a few of which were shown to selectively target into vesicles. However, protein factors, mechanisms, and sequence elements contributing to this specificity remain largely elusive. Here, we use a reporter system that results in different types of modified transcripts to decipher the specificity determinants of RNAs released into EVs. First, we found that small RNAs are more efficiently packaged into EVs than large ones, and second, we determined absolute quantities for several endogenous RNA transcripts in EVs (U6 snRNA, U1 snRNA, Y1 RNA, and GAPDH mRNA). We show that RNA polymerase III (pol III) transcripts are more efficiently secreted into EVs compared to pol II-derived transcripts. Surprisingly, our quantitative analysis revealed no RNA accumulation in the vesicles relative to the total cellular levels, based on both overexpressed reporter transcripts and endogenous RNAs. RNA appears to be EV-associated only at low copy numbers, ranging between 0.02 and 1 molecule per EV. This RNA association may reflect internal EV encapsulation or a less tightly bound state at the vesicle surface.

## 1. Introduction

Extracellular vesicles (EVs) are omnipresent lipid particles released by nearly every cell and are involved in cell–cell communication [1,2,3]. As this already implies, EVs are not simply empty lipid bins, but rather represent transport vesicles, which are associated with various biomolecules. Although the composition of EVs is a highly debated topic, a large number of different biomolecules have been identified to be associated with EVs [4,5,6]. However, the presence or absence of certain molecules appears strongly dependent on the starting material, the type of purification, and the research group [7,8]. Nevertheless, various proteins (e.g., receptors, transcription factors, enzymes, and extracellular matrix proteins), lipids, and nucleic acids (DNA and RNA) have been identified [9,10,11]. In particular, ribonucleic acids have gained considerable attention in the last few years. While most studies focus on the functionally well-characterized miRNAs [12,13,14], the advent of the RNA-Seq technologies allowed to analyze extremely low quantities of RNA, revealing different types of EV-associated RNA. Several studies described that, in addition to the already mentioned miRNAs, other non-coding RNAs, such as lncRNAs, snoRNAs, tRNAs, rRNAs, piRNAs, Y RNAs, and circRNAs are also present [15,16,17,18,19]. In some reports, the existence of mRNAs within vesicles could also be demonstrated, transferable to and functionally active in recipient cells [20,21,22].

Apart from the identification of distinct RNA classes, the question of a specific and direct loading into EVs has remained unanswered. Some mechanisms have been proposed, such as protein-mediated secretion of RNAs, in which RNA-binding proteins assist with selective release of RNAs to EVs, some through recognition of RNA-binding motifs, in particular for miRNAs [23,24,25,26,27]. In addition to protein-mediated exports, less selective export mechanisms and determinants have also been identified, such as size dependence, whereby mainly small RNAs are present in vesicles; a concentration-directed RNA sorting into EVs was also considered [19,28,29]. Interestingly, recent quantitative studies showed that extracellular miRNAs could be relatively rare in EVs, with a range between 0.0001 and 0.1 molecules per EV [30,31,32]. Likewise, detected mRNAs are less frequently associated with EVs. In contrast, other RNAs, in particular those derived from RNA Polymerase III, such as Y RNAs and tRNA (fragments) as well as snRNA and rRNA fragments, appear to be more abundant in EVs [31]. The previous assessment that miRNAs are enriched in EVs may be related to the fact that miRNAs are predominantly associated with RNA-binding proteins, such as AGO2, and are found as contaminants during isolation or associated with the outside surface of EVs, thus protecting extracellular miRNAs from degradation by RNases [28,33,34,35].

Here, we used different reporter expression constructs to determine which RNA characteristics mediate the preferential release of RNAs into vesicles. In addition to a length dependence, we were able to show that RNA polymerase III transcripts are preferentially associated with EVs. In addition, we did absolute quantification of various endogenous RNAs in EVs versus total cells. In sum, EV-mediated RNA secretion appears to be inefficient and only a few RNA molecules are present within each vesicle. Our results and EV-RNA quantitation should contribute to our mechanistic understanding of EV-mediated transfer and functional range of RNAs.

## 2. Materials and Methods

### 2.1. Cell Culture and Transfection

All cell lines A549, ES-2, HEK293, HepG2, and U373 were maintained at 37 °C in 5% CO_2_ in Dulbecco’s Modified Eagle Medium (Gibco™, Thermo Fisher Scientific, Waltham, MA, USA) supplemented with 10% fetal bovine serum (Gibco™, Life Technologies, Carlsbad, CA, USA). For EV production, between 8 × 10^6^ and 2 × 10^7^ cells were seeded per T175 flask (Greiner Bio-One GmbH, Frickenhausen, Germany), depending on the cell line. After 24 h, the medium was changed to CD293 medium (Gibco™, Thermo Fisher Scientific) supplemented with 1% penicillin-streptomycin and 1x GlutaMAX™ (Gibco™, Thermo Fisher Scientific), followed by cell culture for 48 h. To generate EV-depleted FBS, FBS-derived EVs were pelleted by ultracentrifugation at 110,000× *g* at 4 °C for 18 h. The EV-depleted supernatant was mixed with DMEM to obtain a final concentration of 5%.

For transfection, 5–6 × 10^6^ cells per 15-cm dish (Greiner Bio-One GmbH) were seeded in DMEM supplemented with 10% FBS one day before transfection. Transfection was performed by TurboFect (Thermo Fisher Scientific Waltham, MA, USA), using 20 µg plasmid per plate. After 4 h, the medium was changed to DMEM supplemented with 5% EV-depleted FBS, and cells were cultured for 24 h.

### 2.2. Isolation of Extracellular Vesicles

EVs were isolated from the cell culture medium by differential centrifugation, first at 2000× *g* for 10 min, and second, at 5000× *g* for 5 min at 4 °C. Supernatants (200 mL) were filtered through a 0.22 µm pore filter (ROTILABO^®^ CME, Carl Roth, Karlsruhe, Germany), followed by ultrafiltration through centrifugal filters (Amicon Ultra-15, 10 kDa, Merck KGaA, Darmstadt, Germany) to a final volume of 500 µL. Subsequently, the concentrated EV samples were subjected to size exclusion chromatography using qEVoriginal 70-nm columns (IZON Science Ltd., Christchurch, New Zealand). The EV-containing fractions were pooled and subjected to a final ultracentrifugation step at 110,000× *g* at 4 °C for two h (Beckman Coulter, Krefeld, Germany; MAX-XP centrifuge, TLA-100.4 rotor). Finally, the EV pellet was resuspended in filtered 1 × PBS.

### 2.3. SDS-PAGE and Western Blot Analysis

The 1 × 10^9^ EVs were lysed with 2 × SDS sample buffer (for CD63 samples, non-reducing conditions were used) and run on an SDS-12% polyacrylamide protein gel. Proteins were transferred to a nitrocellulose membrane (Bio-Rad Laboratories GmbH, Feldkirchen, Germany) and membranes were blocked with 5% (*w*/*v*) milk powder (Carl Roth) in phosphate-buffered saline supplemented with 0.05% Tween (PBS-T) for one hour. Subsequently, membranes were probed using the following antibodies: mouse anti-human CD63 (1:500, Life Technologies; TS63), rabbit anti-human Alix (1:1000, Abcam, Cambridge, UK; EPR15314), and rat anti-human Flotillin-1 (1:1000, BioLegend, Koblenz, Germany; W16108A) overnight at 4 °C in 5% milk in PBS-T. The blot was washed three times with PBS-T and then incubated with HRP-conjugated anti-mouse, anti-rabbit, and anti-rat secondary antibodies (Sigma-Aldrich, Munich, Germany), respectively, for one hour at room temperature. After washing three times with PBS-T, the blots were incubated for five minutes at room temperature with Lumi-Light Western Blotting Substrate (Roche, Basel, Switzerland) and visualized with Amersham Hyperfilm^TM^ ECL (GE Healthcare, Freiburg, Germany).

### 2.4. Electron Microscopy

EVs were prepared for electron microscopy as described previously [36] with few modifications: Purified EVs were fixed with an equal amount of 4% PFA. A small volume (5–7 µL) of this suspension was transferred to a formvar/carbon-coated meshed copper grid (Ted Pella Inc., Redding, CA, USA) and air-dried for 20 min. The grids were washed with sterile-filtered PBS and fixed for 5 min with 1% glutaraldehyde. After 8 washing steps (2 min each) with distilled water, the sample was incubated with 1% uranyl acetate for 5 min and then transferred to 2% methylcellulose supplemented with 4% uranyl acetate (ratio 9:1) and incubated for 10 min on ice. Excess fluid was removed, and the grid was air-dried for up to 10 min. Images were taken with a Zeiss EM 900 (Zeiss, Jena, Germany) at 80 kV equipped with a 2k slow-scan CCD camera (TRS-STAR GmbH, Stutensee, Germany).

### 2.5. Nano-Flow Cytometry (NanoFCM)

For NanoFCM, a Nano Analyzer (NanoFCM Co., Ltd., Nottingham, UK) equipped with a 488 nm laser, was calibrated using 200 nm polystyrene beads (NanoFCM Co.) with a defined concentration of 2.08 × 10^8^ particles/mL, which were also used as a reference for particle concentration. In addition, monodisperse silica beads (NanoFCM Co. Ltd.) of four different sizes served as size reference standards to calibrate the size of EVs. Freshly filtered (0.22 µm) 1 × PBS was analyzed as background signal and subtracted from the other measurements. Each distribution histogram or dot plot was derived from data collected for one minute with a sample pressure of 1.0 kPa. The EV samples were diluted with filtered (0.1 µm) 1 × PBS, resulting in a particle count in the optimal range of 2500–12,000 events. Particle concentration and size distribution were calculated using the NanoFCM software (NF Profession V1.08, NanoFCM Co. Ltd.).

For immunofluorescence staining, the following antibodies were used (BioLegend): FITC-conjugated mouse anti-human CD9 antibody (clone HI9a), FITC-conjugated mouse anti-human CD81 antibody (clone TAPA-1), and PE-conjugated mouse anti-human CD63 antibody (clone H5C6); as isotype controls, FITC-conjugated mouse IgG1, κ (clone MOCP-21), and PE-conjugated mouse IgG2a, κ (MOPC-173), 2 ng/µL of each antibody in 100 µL 1 × PBS. After removing antibody aggregates by centrifugation at 12,000× *g* for 10 min, the supernatant was added to 2 × 10^8^ purified EVs, followed by incubation overnight at 4 °C under constant shaking and washing with 1 mL 1x PBS by ultracentrifugation at 110,000× *g* for 60 min at 4 °C (Beckman Coulter MAX-XP centrifuge, TLA-145 rotor). The pellet was resuspended in 50 μL 1 × PBS for NanoFCM analysis.

### 2.6. RNA Isolation, Relative and Absolute qPCR

RNA was isolated using the Total RNA Purification Kit (Norgen BioTek Corp. Thorold, ON, Canada), followed by analysis of RNA concentration and integrity (Agilent 2100 Bioanalyzer; RNA 6000 Pico Kit, Agilent Technologies, Santa Clara, CA, USA). For RT-PCR, 1 ng of total EV-RNA and 1000 ng of total cellular RNA were used for cDNA synthesis (qScript™ Flex cDNA synthesis kit, Quantabio, Berverly, MA, USA), with primers specific for the constant region of the reporter constructs. All primers were designed using primer3 version 4.0 (https://primer3.ut.ee/; accessed on 30 September 2021). All oligonucleotides can be found in Table S1. RT-PCR products were analyzed by 2% agarose gel electrophoresis. For absolute and relative quantification, 2 ng total EV RNA and 1000 ng total cellular RNA were reverse-transcribed, using qScript cDNA synthesis kit (Quantabio). Real-time PCR was carried out on an Eppendorf realplex2 thermocycler (Luna^®^ Universal qPCR Master MIX; New England Biolabs, Ipswich, MA USA). Relative expression levels of transcripts in cells and EVs were calculated by the ΔΔCt-method, with each target normalized to Y1 and vault RNA [37]. Unless otherwise stated, three replicates were used to calculate standard deviations. The relative levels of cellular versus EV transcripts were determined (2^−ΔΔCt^) and normalized to the geometric mean of two housekeeping genes. For absolute quantification, RNA standards were generated by in vitro transcription (HiScribe™T7 High Yield RNA Synthesis Kit, New England Biolabs), based on templates carrying the T7 promoter and the sequences of U6 snRNA, Y1 RNA and U1 snRNA, respectively. Moreover, 50 ng of each standard was reverse-transcribed (qScript cDNA synthesis Kit; Quantabio) and diluted in six 10-fold steps (50 ng to 5 fg). Based on this, a standard curve was calculated with three replicates for standard deviations. Absolute concentrations of the samples were determined using the following equation:

$$Y\;molecules/µL=\frac{X\;RNA\;g/µL}{\left(transcript\;length\;\left[nts\right]*321.47+18.02\right)*6.022*10^{23}}$$

For Xrn1 degradation assays, 200 ng total RNA from EVs and cells were digested with 2 units of Xrn1 (New England Biolabs) for one hour at 37 °C. RNA was purified using Monarch^®^ RNA Cleanup Kit, followed by reverse transcription (qScript cDNA synthesis kit; Quantabio) and PCR, using primers against GAPDH, U6 snRNA and Y1 RNA. For standard agarose gel electrophoresis of RT-PCR products, DNA marker was used GeneRuler Ladder Mix with 500, 400, 300, 200, and 100 bp (Thermo Fisher Scientific).

### 2.7. Plasmid Constructs

Pol II/poly(A), pol II/U1-3′ box, und pol III/U6-term: based on pCDNA3 as vector, RNA expression constructs were designed and synthesized (GeneArt, Thermo Fisher Scientific), each containing the same random-generated sequence of 40 bp and unique flanking constant regions (20 bp each). The insert for Pol II/U1-3′ box additionally contained the sequence of the U1-3′-box at the 3′ end. Both inserts were flanked by restriction sites for *Hin*dIII at the 5′- and *Xba*1 at the 3′ end. For the Pol III/U6-term construct, first, the CMV promoter in pcDNA3.1 was deleted and replaced by a synthetic insert (GeneArt, Thermo Fisher Scientific) consisting of the U6 promoter, a multiple cloning site, and the U6 termination signal (pcDNA3_U6). Then a synthetic fragment consisting of the random-generated 40-bp sequence and the unique flanking constant regions was inserted (see above). For sequences, see Table S1.

Length constructs (80,120,200,360,680): Based on the pcDNA3_U6 vector (see above), a series of constructs with inserts of different lengths was derived. The synthetic inserts (GeneArt, Thermo Fisher Scientific) consist of two 40-bp sequences of multiple copies thereof, inserted between the *Hin*dIII and *Xba*I sites and generating length constructs 80, 120, 200, 360, 680.

### 2.8. Northern Blot

For glyoxal Northern blot analysis, 300 ng of both total RNA from cells and the corresponding EV-RNA (isolated from 2.4 × 10^6^ cells/mL and 350 mL supernatant) was mixed with glyoxal loading dye (Ambion) and incubated at 50 °C for 30 min. RNA was separated by agarose gel electrophoresis (1.2% in 1 × MOPS buffer). The gel was transferred onto a nylon membrane by semidry blotting followed by RNA crosslinking using UV-light (254 nm; 120 J/cm^2^) and probed with a single-stranded RNA probe (DIG RNA Labeling Mix; Roche), directed against GAPDH mRNA. Hybridization was performed in NorthernMax buffer (Thermo Fisher Scientific), and washes of the blot and probe detection with alkaline phosphatase-conjugated anti-DIG-Fab fragments were done as described in the Roche manual (Roche).

## 3. Results

### 3.1. Isolation and Characterization of Extracellular Vesicles

**Figure 1:**
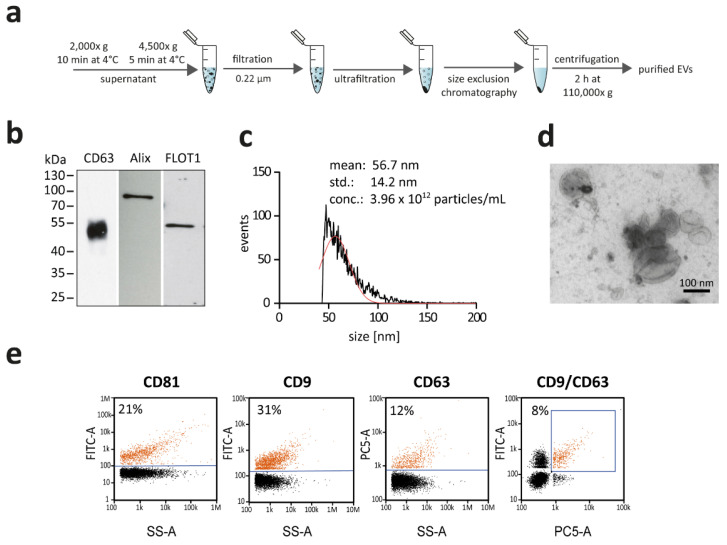
Isolation and characterization of extracellular vesicles from HEK293 cells. (**a**) Schematic overview of EV purification from cell culture supernatant. EVs were purified by a combination of differential centrifugation steps, followed by ultrafiltration and size exclusion chromatography. The EV-containing fractions were pooled and subjected to a final ultracentrifugation step. Subsequently, the EVs were resuspended in PBS. (**b**) Western blot analysis of EV-marker proteins CD63, ALIX, FLOT1. (**c**) Nano-flow cytometry (NanoFCM) analysis, depicting the size distribution (diameter) and concentration of particles and indicating mean and standard deviation (std.) of the size distribution. (**d**) Electron microscopy of purified vesicles. Purified EVs were counterstained by uranyl acetate. (**e**) Single-particle phenotyping of HEK293-derived EVs. EVs were fluorescently labelled with FITC-conjugated antibodies specific to CD81 and CD9 or PE-conjugated CD63. Bivariate dot-plots of indicated fluorescence versus SSC are shown. In addition, double positives for CD9/CD63 are depicted.

In order to characterize the selective release of RNAs into EVs using our reporter system, we isolated EVs from HEK293 cell culture by a combination of differential centrifugation, ultrafiltration, size exclusion chromatography, and a final ultracentrifugation step (Figure 1a). 

The isolated EVs were characterized, according to minimal information for studies of extracellular vesicles (MISEV) 2018 guidelines [8]. First, EV samples were analyzed by immunoblotting, using antibodies detecting one of the “classical” tetraspanin markers, CD63, as well as two intravesicular markers, ALIX and FLOT1 (flotillin-1) (Figure 1b). Second, we analyzed EV size distribution by nano-flow cytometry (NanoFCM), which detected particles with a mean of approximately 57 nm in diameter, consistent with the established size of small EVs (Figure 1c). In addition, we assessed the specific enrichment of EVs using transmission electron microscopy (Figure 1d).

To obtain a more detailed view of our isolated samples, we also conducted single-particle analysis by NanoFCM (Figure 1e). The three tetraspanin markers CD81, CD9, and CD63 were used for staining, in which 21% of all measured particles were positive for CD81. Furthermore, we performed co-staining of CD9 and the exosomal marker CD63. Both CD9 and CD63 showed similar staining efficiencies with 31% and 12%, respectively. When analyzing the double-positive events, 8% of all particles stained positive.

### 3.2. RNA Polymerase III Transcripts Are Preferentially Loaded into EVs

**Figure 2:**
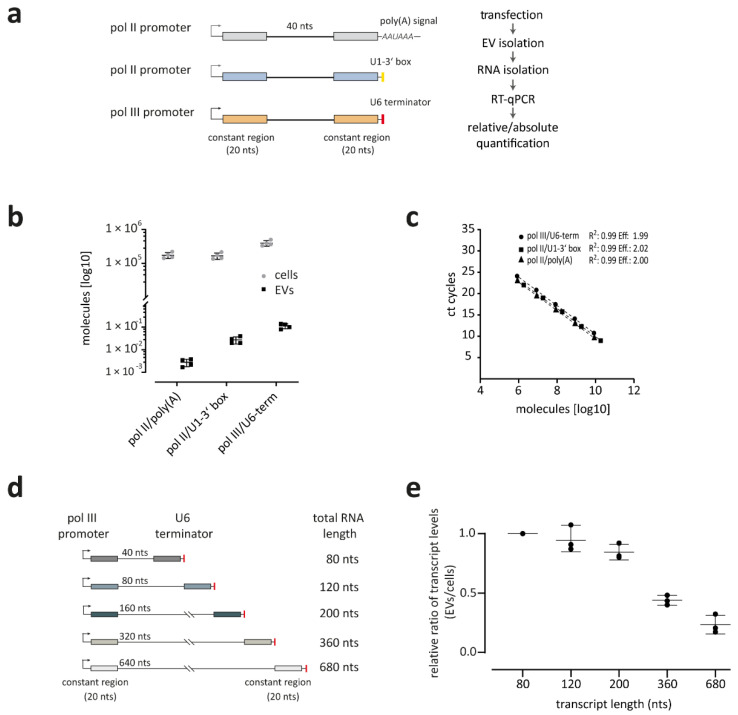
RNA polymerase expression platforms affect RNA packaging into extracellular vesicles. (**a**) Design of expression constructs: pol II/poly(A), pol II/U1-3′ box, and pol III/U6-term. After co-transfection of all three constructs, total RNA was isolated from HEK293 cells and purified EVs, followed by RT-PCR, using primers directed against the respective constant regions. (**b**,**c**) Expression-dependent efficiency of RNA packaging into EVs, quantified by RT-qPCR in absolute copy numbers (molecules per cell and per EV; in log10; panel. For the determination of absolute values, four datasets were used (*n* = 4), indicating mean and standard deviations (for standard curve with equations and amplification efficiencies, see panel **c**). (**d**,**e**) Size-dependent packaging of RNAs into extracellular vesicles. Design of expression constructs. All constructs harbor an RNA pol III (U6) promotor, followed by a randomly generated sequence with different sizes (40, 80, 160, 320, 640 nts), flanked by two unique constant regions (20 nts). All constructs contain a U6 termination signal (red), resulting in an RNA with a γ-monomethyl cap and no poly(A) tail (panel **d**). Length-dependent efficiency of RNA packaging into EVs, quantified by RT-qPCR as a relative ratio between EV-associated and total cellular RNA (EV/cells). Constructs were transfected in HEK293 cells, followed by total RNA isolation from cells and purified EVs. RT-qPCR was performed and the relative packaging of the individual transcripts (80, 120, 200, 360, 680 nts) into extracellular vesicles was analyzed (*n* = 3), normalized total expression in cells (panel **e**).

When summarizing the various classes of RNAs reported to be associated with EVs, we noticed a clear propensity towards RNA polymerase III transcripts, such as tRNAs (including fragments thereof), Y RNA, vault RNA, and U6 snRNA. To determine more systematically whether RNAs are EV-loaded in an RNA polymerase-dependent manner, we used reporter constructs and compared three different expression platforms, based on three constructs (Figure 2a): all three constructs contain a specific constant region, enabling the individual detection of the transcripts by RT-PCR, as well as a common, random-generated sequence of 40 nts. First, the pol II-poly(A) construct contains an RNA polymerase II (pol II) promoter (CMV) and terminates with a classical poly(A) signal (AAUAAA), generating an mRNA-like transcript. Second, the pol II/U1-3′ box construct harbors the same pol II promoter, but is terminated at the 3′ end by a U1-3′ box, generating a pol II transcript without a poly(A) tail. As a third reporter, a pol III construct was used, which included a U6 snRNA (RNU6-1)-derived pol III promoter as well as a U6 terminator (U5 in the transcript). All three constructs were co-transfected into HEK293 cells and after a 24 h incubation, EVs were purified, and RNA was isolated. Quantification was subsequently performed by absolute RT-qPCR assays (Figure 2b). For an absolute quantification of transcripts, standard curves were generated with three in vitro transcribed RNAs, derived from the reporter constructs, to obtain similar amplification efficiencies to the targets (Figure 2c). In addition to the EV-RNAs, we performed absolute quantification of the overexpressed RNAs in the progenitor cells of the EVs. Inside the cells, we calculated for pol III/U6-term 4.1 × 10^5^, for pol II/U1-3′ box 1.8 × 10^5^, and for pol II/poly(A) 1.8 × 10^5^ molecules per cell, whereas the number of RNA molecules inside EVs was quite low. The most abundant detectable RNA inside EVs was the pol III/U6-term with 0.1 molecules per EV. Both overexpressed pol II/U1-3′ box and pol II/poly(A) RNAs were even less abundant with 0.02 and 0.003 molecules per EV, respectively (Figure 2b).

### 3.3. Smaller Transcripts Are Preferentially Loaded into EVs

Since several studies indicate that smaller RNAs are preferentially associated with EVs, we designed five pol III promoter-based reporter constructs, expressing RNAs of different lengths with a random sequence (80, 120, 200, 360, and 680 nts; Figure 2d), all of them consisting of an individual constant region for detection by specific primers. These constructs were transfected and expressed in HEK293 cells, and the released EVs were isolated after 48 h. All five transcripts could be detected in cells and EVs. To analyze whether EV-mediated RNA release depends on transcript size, we performed RT-qPCR and analyzed the relative levels of transcripts in EVs versus cells (Figure 2e). For an internal normalization, we set the shortest transcript (80 nts) as a reference to 1. Based on the size distributions, we conclude there is a clear inverse relationship between transcript size and EV-mediated RNA release, and that thereby RNA length represents an important determinant.

### 3.4. Absolute Quantification of Endogenous RNAs in EVs

**Figure 3:**
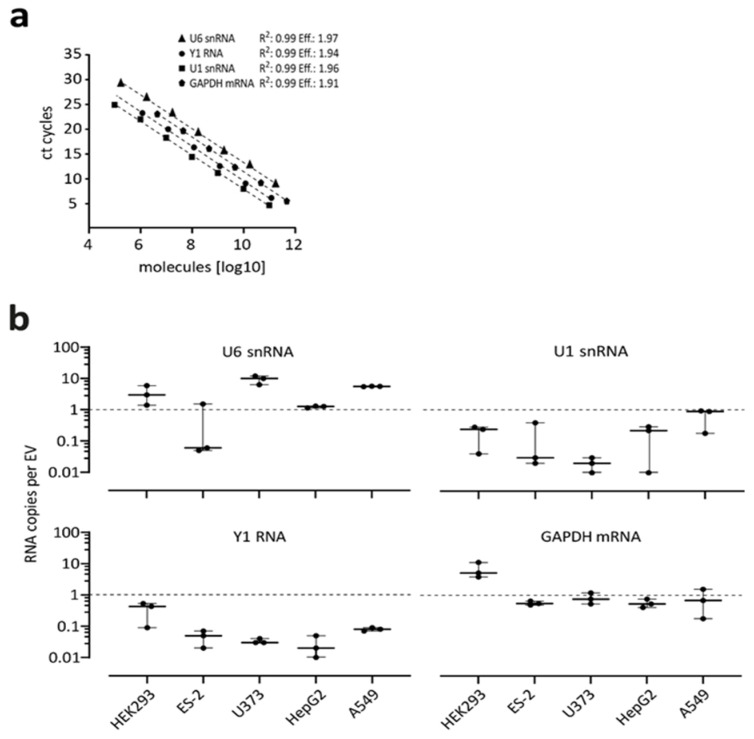
Absolute quantification of endogenous RNAs in EVs: U6 snRNA, Y1 RNA, U1 snRNA, and GAPDH mRNA. (**a**,**b**) Absolute quantification of copy numbers per EV for four different RNAs. For standard curves with equations and amplification efficiencies used for absolute quantification by RT-qPCR, see panel **a**. EVs were purified from five different cell lines (HEK293, ES-2, U373, HepG2, and A549), followed by RT-qPCR assays (panel **b**; copy numbers per EV in log10-scale; *n* = 3, with mean and standard deviations).

Complementary to reporter constructs, which are under the control of strong promoters and express standardized artificial transcripts, we also quantitatively analyzed a set of endogenous RNAs. To cover a more comprehensive set of transcripts, we focused on pol III-derived U6 snRNA and Y1 RNA, as well as on pol II-derived U1 snRNA and GAPDH mRNA. To exclude potentially cell-specific loading differences, we analyzed in parallel EVs from five different cell lines: ES-2 (ovarian clear cell adenocarcinoma), U373 (glioblastoma), HepG2 (hepatocyte carcinoma), A549 (lung adenocarcinoma), in addition to HEK293 cells. Purified EVs of all these cell lines were measured by NanoFCM (Supplementary Figures S1 and S2), followed by RNA isolation (Supplementary Figure S3). Absolute quantification of the individual RNAs was based on standard curves derived from in vitro transcribed standards, with primer amplification efficiencies of 1.97 for U6 snRNA, 1.96 for U1 snRNA, 1.94 for Y1 RNA, and 1.91 for GAPDH mRNA (Figure 3a). Similarly, to the above-mentioned reporter approaches, RNA pol III transcripts were found to be the most abundant in EVs. In particular, the U6 snRNA with 0.7–4.2 molecules per EV was found most frequently in all tested cells. The Y1, as well as the U1 snRNA as RNA Pol II transcript without poly(A) tail, could be detected in copy numbers between 0.2 and 1.9 molecules per EV. Interestingly, GAPDH mRNA as RNA Pol II transcript with a poly(A) tail, could be found in copy numbers between 6.8 and 0.57 molecules per vesicle (Figure 3b).

### 3.5. GAPDH mRNA Shows an Aberrant Cap and Poly(A) Status

**Figure 4:**
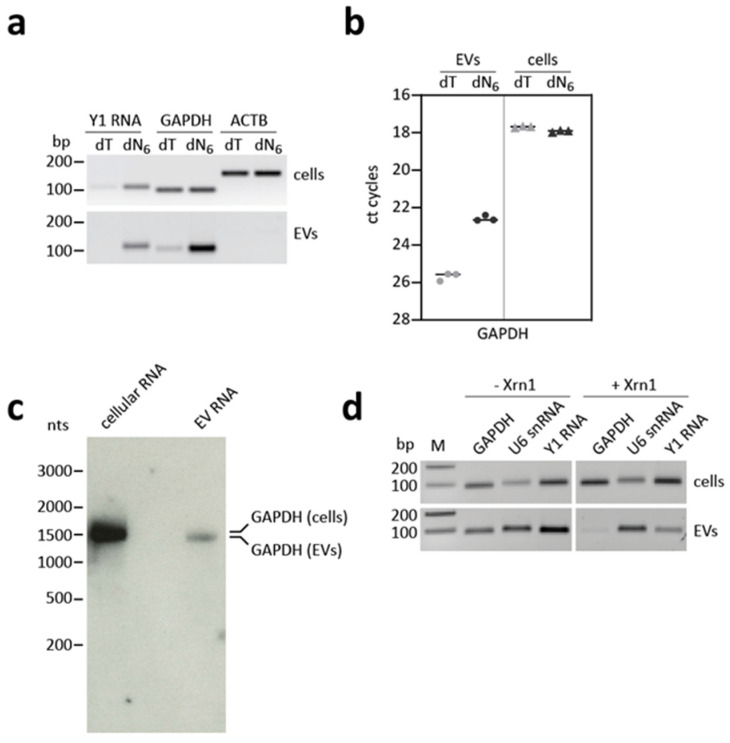
EV-associated and cellular GAPDH mRNA differ in size and polyadenylation status. (**a**) Total RNA from ES-2 cells and their corresponding EVs were isolated, followed by RT-PCR. For reverse transcription, either oligo(dT) or random hexamers (dT, dN_6_) were used. Markers (200, 100 bp). (**b**) RNA was isolated from ES-2 cells and their corresponding EVs. For reverse transcription, either oligo(dT) or random hexamers (dT, dN_6_) were used, and GAPDH mRNA was quantified by RT-qPCR. (**c**) Detection of GAPDH mRNA in EVs by the glyoxal Northern blot analysis. Total cellular and EV-RNA (300 ng) were analyzed by glyoxal agarose gel electrophoresis and Northern blotting. Exposure time 3 min (**d**) Total RNA from ES-2-derived EVs was isolated and treated with Xrn1 exoribonuclease (+Xrn1). As control, no enzyme was added (−Xrn1). RT-PCR was performed using primers directed against transcripts indicated. Markers (200, 100 bp).

The specific characteristics of the individual RNAs within EVs have not yet been fully clarified. In particular, whether mRNAs that are detectable in EVs exist in full-length copies and carry of a functional poly(A) tail, could not yet be verified in sufficient detail. To initially analyze this, we isolated RNA from ES-2 cells and their corresponding EVs, followed by semi-quantitative RT-PCR assays (Figure 4a). For cDNA synthesis, either oligo(dT) or random hexamer primers (dT, dN_6_) were used to evaluate the presence of a poly(A) tail. In the dN_6_ approach, U6 snRNA and Y1 RNA were detectable in cells as well as in EVs, whereas ß-actin (ACTB), could not be detected. However, if cDNA synthesis was performed with oligo(dT) instead, the Y1 RNA signal was strongly reduced, as expected for a non-polyadenylated RNA. In contrast, the signals for GAPDH and ACTB mRNA remained similar in cellular RNA, comparing oligo(dT) and dN_6_ approaches, but the GAPDH signal strongly decreased for the EV-RNA in the oligo(dT) assay. This suggests that GAPDH mRNA exists predominantly in a non-polyadenylated form when EV-associated. To further confirm this quantitatively, we also performed RT-qPCR (Figure 4b), which further supports the results of the semi-quantitative analysis. When using oligo(dT) instead of dN_6_ for the cDNA synthesis, a clear difference in the ct cycle number for EV-associated GAPDH RNA was observed, while no difference was detected in cellular RNA indicating a missing or defective poly(A) tail for EV-associated GAPDH mRNA.

To check whether GAPDH mRNA might be fragmented or shortened in total length in EVs, we used the northern blot analysis to directly visualize GAPDH mRNA. In both cells and EVs, a signal for GAPDH could be detected when the same amount of RNA (300 ng) was used. The GAPDH signal detected in cells corresponds to the expected size of the GAPDH mRNA (approximately 1.6 kb, including 200 nts from the poly(A) tail). However, the signal in EVs appears to be weaker and, furthermore, the band runs slightly below the cellular GAPDH mRNA, which indicates a truncated form (Figure 4c, Supplementary Figure S4). To assay for its 5′-terminal cap, we treated the isolated RNAs with the exoribonuclease Xrn1, which degrades RNAs with a 5′ monophosphate. In contrast, mRNAs with an intact, classical m^7^G cap remain protected from degradation. After Xrn1 treatment, the tested GAPDH mRNA was no longer detectable in the EV fraction, while the signal in the cellular fraction remained. In contrast, U6 snRNA with its γ-monomethyl cap was not a substrate for Xrn1 and, thus, remained stable. The same applied to the Y1 RNA signal (Figure 4d). In sum, our data suggest that the detectable GAPDH mRNA in EVs shows altered characteristics with respect to its 5′- and 3′ modifications. 

## 4. Discussion

Research activities on EV-associated RNAs have recently focused on miRNAs, as they represent suitable candidates for biomarkers and, thus, harbor great potential for the diagnosis and early detection of diseases [38]. In addition, an attractive approach to use EVs for therapeutic purposes involves loading the EVs with specific RNAs [39]. In principle, EVs are considered promising candidates for the clinically relevant transport of biomolecules since they are endogenous, and therefore not as immunogenic as synthetic nanoparticles. However, before EVs can be used in this manner, several remaining issues and open questions need to be addressed [40].

The initial assumption that RNA represents one of the major constituents of EVs has recently been challenged by several quantitative studies. In particular, miRNAs were found as stable extracellular RNAs in non-vesicular fractions [11]. Therefore targeted release of RNAs in EVs represents an important issue; several mechanisms for the selective RNA release have been reported (23–27], although so far no general mechanism has been identified. Since the RNA biogenesis pathways may affect EV-mediated packaging, we compared overexpressed transcripts under pol II and pol III promoter control, as well as with and without poly(A) tail. In addition, since our own previous study on RNA release had suggested the length of endogenous RNAs as an important determinant [19]), we compared overexpressed transcripts of different lengths (80–680 nts), carrying oligomerized copies of the same sequence element. As a result, we found pol III transcripts to be most efficiently EV-released and established a reciprocal relationship between transcript length and release efficiency. Therefore, our results from systematic assays in an overexpression system are consistent with and further specify previous findings based on selected endogenous transcripts or RNA-seq. These findings are also reflected by the analysis of endogenous RNAs from EVs, which are for the most part smaller than 200 nts (Figure S3).

Why are small RNAs preferentially secreted? On the one hand, it may represent a disposal pathway whereby RNA fragments are packaged into EVs, or on the other hand, it may be explained by the fact that there is simply not enough space within the vesicle for larger RNA molecules. Assuming that an RNA of approximately 1000 nts can be organized in an ellipsoidal structure of approximately 40 nm in diameter [41] and a size of 40–100 nm for small EVs, based on close packing of equal spheres we would assume that 0.7 to 11 molecules can be loaded per vesicle. Finally, one should not forget that this calculation refers to naked RNA not associated with proteins.

At the same time, we were able to clearly show that the RNA-loading capacity of EVs appears to be limited to a surprisingly low number of molecules, based on measuring absolute copy numbers of specific RNA transcripts, both after overexpression of transcripts and for endogenous RNAs (U6 and U1 snRNAs, Y1 RNA, GAPDH mRNA), as well as by comparing EVs from five different cell lines. Since this limits the application of EVs as effective cargo vehicles, it would be useful to evaluate also expression platforms other than CMV- or U6 promoter-based ones, or other cell lines, as alternatives.

Complementary to artificial transcripts generated by different expression platforms, we quantitatively compared EV packaging of a set of natural RNAs that represent different biogenesis pathways resulting in different terminal modifications: Y1 RNA, U1, U6 snRNA, and GAPDH mRNA. Y1 RNA and U6 snRNA are Pol III-derived, both exhibiting different 5′ modifications (triphosphate for Y1 RNA, γ-monomethyl-triphosphate for U6 snRNA [42,43]. U1 snRNA is a small non-polyadenylated Pol II transcript, and GAPDH a classical mRNA. As a result, we found that even the enriched, naturally occurring small RNAs exist in rather low copy numbers in EVs, ranging between 0.02 (U1 snRNA/U373 cells) and 9 molecules per EV (U6 snRNA/U373 cells), with U6 snRNA being relatively most abundant in all cell lines tested. This appears to be in accordance with a study by Wei et al. [31], where similar numbers were reported based on a stoichiometric estimation of the EV-associated RNAs. In addition, we have not identified dramatic variations between the five cell lines assayed. It is worth noting that due to its abundance and consistent occurrence, the U6 snRNA qualifies as a candidate for a housekeeping EV-RNA.

Interestingly, we detected the GAPDH mRNA at surprisingly high levels of approximately one molecule per EV in all cell lines tested. This appears to be contradictory to what we have shown above, as the GAPDH mRNA with its 1.4 kb length represents the longest transcript we have examined. However, our RT-qPCR quantification did not assess the mRNA integrity. Instead, sequencing data of EV-associated RNAs provide further evidence for RNA fragments, particularly with respect to mRNAs [16,22]. However, our approaches indicate that the mRNA is present as a truncated version but not as detectable fragments. We would like to emphasize that probably are other sorting and selection factors in addition to size and the biogenesis pathway of the individual RNAs and here in particular GAPDH mRNA may exist, as already shown for several RNAs [23,24,25,26,27]. Further methodological approaches such as sequencing of the entire transcript would be necessary to finally clarify this question but are beyond the scope of this study.

In addition, our approaches for examining the 5′ and 3′ status of mRNA suggest that GAPDH mRNA may exist as a mature mRNA, but rather lacks the mRNA-typical cap and part of or the entire poly(A) tail. Because of this limitation to two mRNAs, we cannot exclude that there may indeed be a transfer of a set of translatable mRNAs into EVs, as shown in several studies [21,44,45].

## Data Availability

Data is contained within the article or supplementary material.

## Supplementary Materials

The following are available online at https://www.mdpi.com/article/10.3390/cells10102674/s1, Figure S1: Size distribution (diameter) and concentration of EVs from five different cell lines; Figure S2: Single-particle phenotyping of EVs isolated from five different cell lines; Figure S3: Profiles of EV-derived RNA from five different cell lines. Figure S4: Detection of GAPDH mRNA in EVs by glyoxal Northern blot analysis. Table S1: DNA-oligonucleotides and primers.
