# Supplementary material for: Molecular Determinants for RNA Release into Extracellular Vesicles
Source: Cells. 2021 Oct 6;10(10):2674. doi: 10.3390/cells10102674 (PMC8534350; doi:10.3390/cells10102674)
Supplement: Supplementary file 1 [file cells-10-02674-s001.zip › cells-1386619-supplementary.pdf]

Supplementary Figure S1

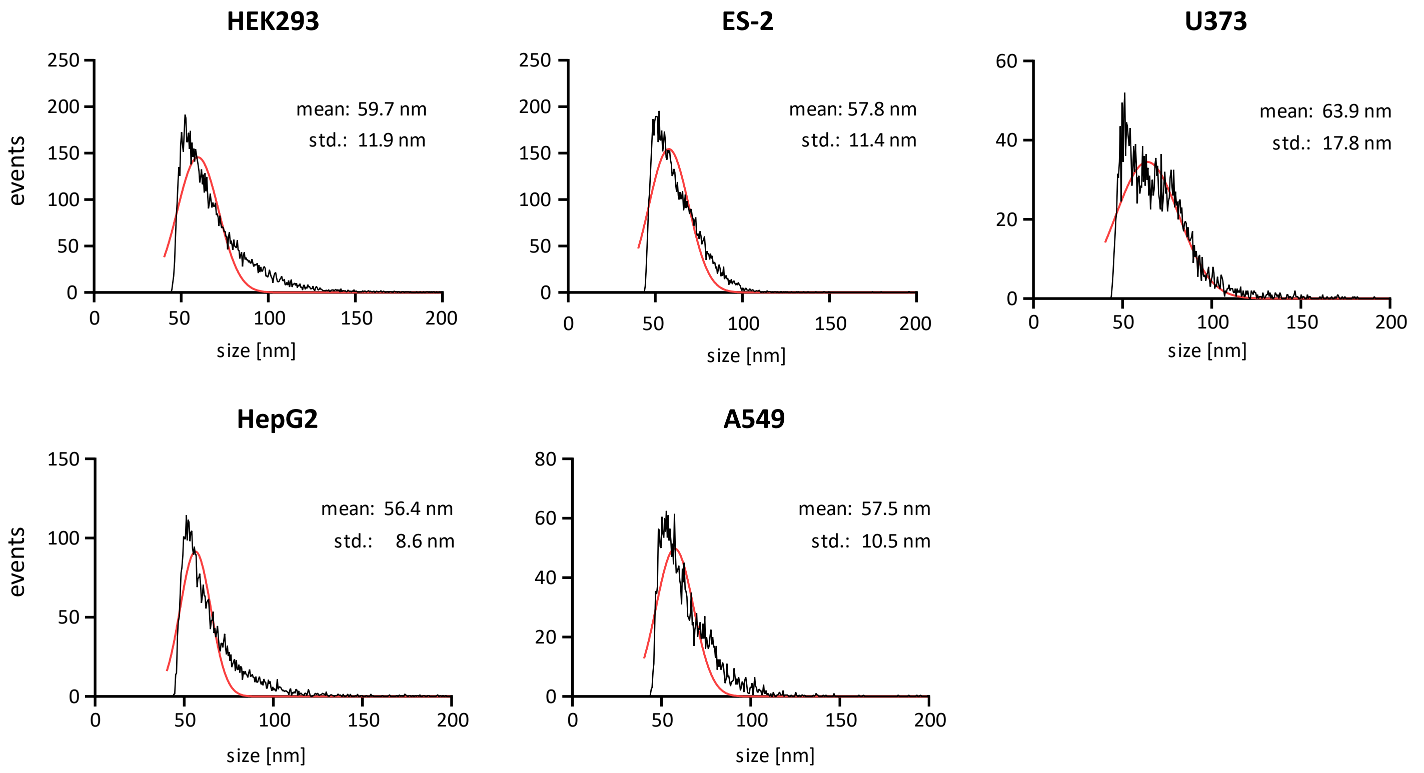

Figure S1. Size distribution (diameter) and concentration of EVs from five different cell lines. Measured by Nano flow cytometry (NanoFCM)

Supplementary Figure S2

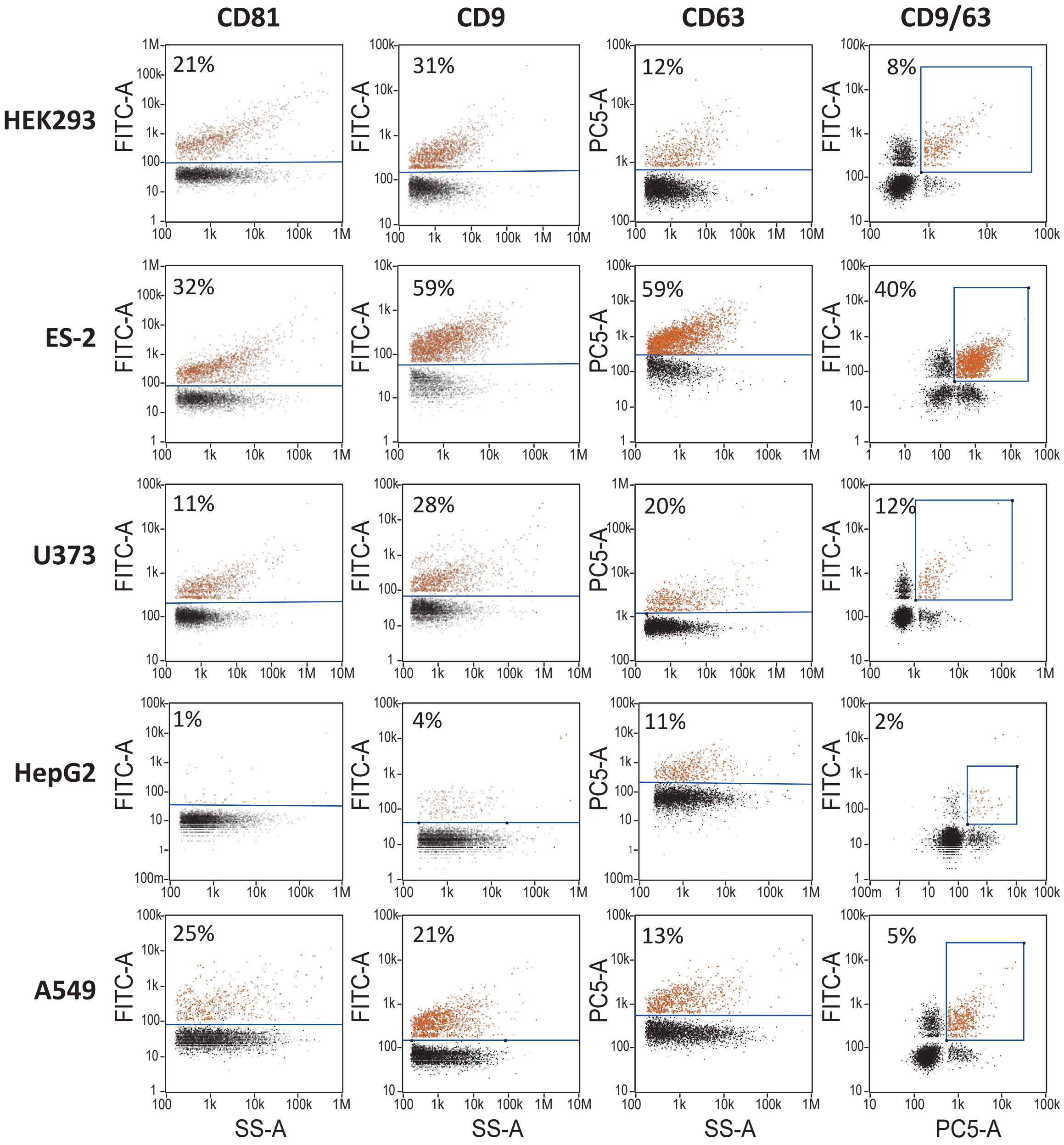

Figure S2. Single-particle phenotyping of EVs isolated from five different cell lines. EVs were fluorescently labeled with FITC-conjugated antibodies specific to CD9 and CD81 or PE-conjugated CD63. Bivariate dot-plots of indicated fluorescence versus SSC are shown. In addition, double positives for CD9/CD63 are depicted on the right-hand side.

Supplementary Figure S3

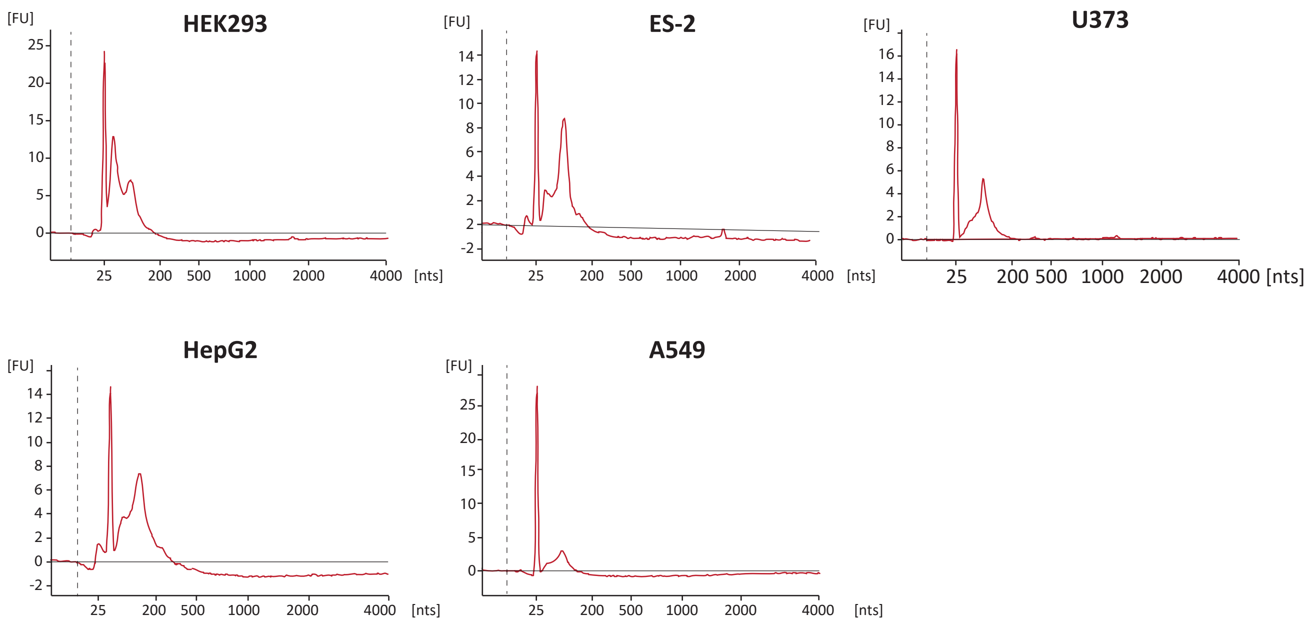

Figure S3. Profile of EV-derived RNA from five different cell lines. Measured with the Bioanalyzer using the Agilent RNA 6000 Pico Kit.

Supplementary Figure S4

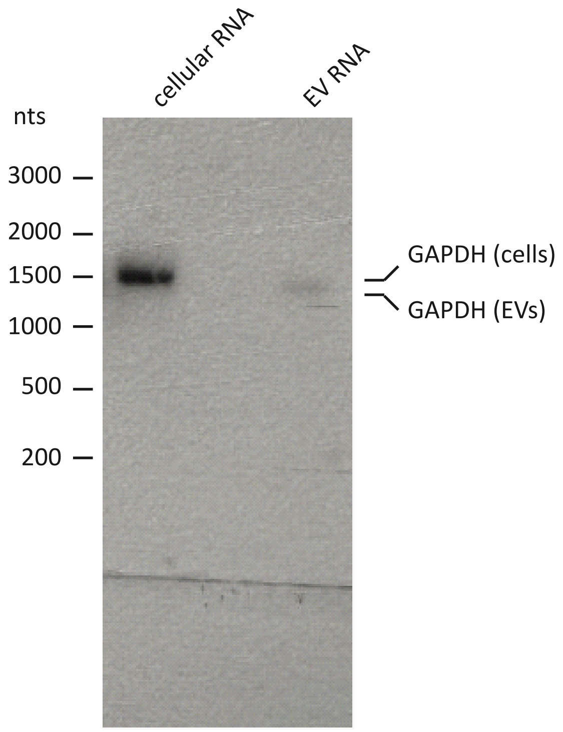

Figure S4. Detection of GAPDH mRNA in EVs by glyoxal Northern blot analysis. Total cellular and EV-RNA (300 ng) were analyzed by glyoxal agarose gel electrophoresis and Northern blotting. One minute exposure time.

Supplementary Table S1

RT-qPCR primers for housekeeping gene detection

Name	***Sequence 5' to 3'***
GAPDH fw	TGCACCACCAACTGCTTAGC
GAPDH rev	GGCATGGACTGTGGTCATGAG
ACTB fw	CTGGAACGGTGAAGGTGACA
ACTB rev	AAGGGACTTCCTGTAACAACGCA
U1 snRNA fw	GCGAGGCTTATCCATTGCAC
U1 snRNA rev	GCAGTCCCCCACTACCACAA
U6 snRNA fw	CTCGCTTCGGCAGCACATA
U6 snRNA rev	GCTTCACGAATTTGCGTGTCA
YRNA 1 fw	GGCTGGTCCGAAGGTAGTGA
YRNA 1 rev	GCAGTAGTGAGAAGGGGGGA

DNA-oligonucleotides used for *in vitro* transcription for absolut quantification standards (T7 promoter underlined)

Name	***Sequence 5' to 3'***
Std_GAPDH fw	TAATACGACTCACTATAGGGTGCACCACCAACTGCTTAGCACCCCTGGCCAAGGTCATCCATGACAACTTTGGTATCGTGGAAGGACTCATGACCACAGTCCATGCC
Std_GAPDH rev	GGCATGGACTGTGGTCATGAGTCCTTCCACGATACCAAAGTTGTCATGGATGACCTTGGCCAGGGGTGCTAAGCAGTTGGTGGTGCACCCTATAGTGAGTCGTATTA
Std_U1 fw	TAATACGACTCACTATAGGGGCGAGGCTTATCCATTGCACTCCGGATGTGCTGACCCCTGCGATTTCCCCAAATGTGGGAAACTCGACTGCATAATTTGTGGTAGTGGGGGACTGC
Std_U1 rev	GCAGTCCCCCACTACCACAAATTATGCAGTCGAGTTTCCCACATTTGGGGAAATCGCAGGGGTCAGCACATCCGGAGTGCAATGGATAAGCCTCGCCCCTATAGTGAGTCGTATTA
Std_U6 fw	TAATACGACTCACTATAGGGCTCGCTTCGGCAGCACATATACTAAAATTGGAACGATACAGAGAAGATTAGCATGGCCCCTGCGCAAGGATGACACGCAAATTCGTGAAGC
Std_U6 rev	GCTTCACGAATTTGCGTGTCATCCTTGCGCAGGGGCCATGCTAATCTTCTCTGTATCGTTCCAATTTTAGTATATGTGCTGCCGAAGCGAGCCCTATAGTGAGTCGTATTA
Std_Y1 fw	TAATACGACTCACTATAGGGGGCTGGTCCGAAGGTAGTGAGTTATCTCAATTGATTGTTCACAGTCAGTTACAGATCGAACTCCTTGTTCTACTCTTTCCCCCCTTCTCACTACTGC
Std_Y1 rev	GCAGTAGTGAGAAGGGGGGAAAGAGTAGAACAAGGAGTTCGATCTGTAACTGACTGTGAACAATCAATTGAGATAACTCACTACCTTCGGACCAGCCCCCTATAGTGAGTCGTATTA
Std_PolIII-U6term fw	TAATACGACTCACTATAGGGTGCAATCGGATCGCACTTTTCTCTCTAATGACGTGTTCTTGCCTCAGCTCGATTCGGTAGCACCGCAGAGATTGTCCCTT
Std_PolIII-U6term rev	AAGGGACAATCTCTGCGGTGCTACCGAATCGAGCTGAGGCAAGAACACGTCATTAGAGAGAAAAGTGCGATCCGATTGCACCCTATAGTGAGTCGTATTA
Std_PolII-polyA fw	TAATACGACTCACTATAGGGCGGCTTCAGTTACTTCGCTTCTCTCTAATGACGTGTTCTTGCCTCAGCTCGATTCGGTAGGCTTGTTACAATTGGGCGGA
Std_PolII-polyA rev	TCCGCCCAATTGTAACAAGCCTACCGAATCGAGCTGAGGCAAGAACACGTCATTAGAGAGAAGCGAAGTAACTGAAGCCGCCCTATAGTGAGTCGTATTA
Std_PolII-U1box fw	TAATACGACTCACTATAGGGGTAGGAGGATAGCGGTCGACCTCTCTAATGACGTGTTCTTGCCTCAGCTCGATTCGGTAGTAAAGCCTCGCAGTTCCGA
Std_PolII-U1box rev	TCGGAACTGCGAGGCTTTACTACCGAATCGAGCTGAGGCAAGAACACGTCATTAGAGAGGTCGACCGCTATCCTCCTACCCCTATAGTGAGTCGTATTA

DNA-oligonucleotides used for cloning expression constructs (restrictions sites underlined)

Name	***Sequence 5' to 3'***
U6_prom_*Mlu* I fw	AGTACGCGTGAGGGCCTATTTCCCATGATTCCTTCATATTTGCATATACGATACAAGGCTGTTAGAGAGATAATTAGAATTAATTTGACTGTAAACACAAAGATATTAGTACAAAATACGTGACGTAGAAAGTAATAATTTCTTGGGTAGTTTGCAGTTTTAAAATTATGTTTTAAAATGGACTATCAT<br>ATGCTTACCGTAACTTGAAAGTATTTCGATTTCTTGGCTTTATATATCTTGTGGAAAGGACGAAAGCTTGGTACCGAGCTCGGATCCACTAGTAACGGCCGCCAGTGTGCTGGAATTCTGCAGATATCCATCACACTGGCGGCCGCTCGAGCATGCATCTAGAGGGCCTTTTTTTTGGGCCCAGT
U6_prom_*Apa* I rev	ACTGGGCCCAAAAAAAAGGCCCTCTAGATGCATGCTCGAGCGGCCGCCAGTGTGATGGATATCTGCAGAATTCCAGCACACTGGCGGCCGTTACTAGTGGATCCGAGCTCGGTACCAAGCTTTTCGTCCTTTCCACAAGATATATAAAGCCAAGAAATCGAAATACTTTCAAGTTACGGTAAGCATATG<br>ATAGTCCATTTTAAAACATAATTTTAAAACTGCAAACTACCCAAGAAATTATTACTTTCTACGTCACGTATTTTGTACTAATATCTTTGTGTTTACAGTCAAATTAATTCTAATTATCTCTCTAACAGCCTTGTATCGTATATGCAAATATGAAGGAATCATGGGAAATAGGCCCTCACGCGTACT
PolIII/U6term_*Hin* dIII fw	AAGCTTTGCAATCGGATCGCACTTTTCTCTCTAATGACGTGTTCTTGCCTCAGCTCGATTCGGTAGCACCGCAGAGATTGTCCCTTTCTAGA
PolIII/U6term_*Xba* I rev	TCTAGAAAGGGACAATCTCTGCGGTGCTACCGAATCGAGCTGAGGCAAGAACACGTCATTAGAGAGAAAAGTGCGATCCGATTGCAAAGCTT
PolII/polyA_*Hin* dIII fw	AAGCTTCGGCTTCAGTTACTTCGCTTCTCTCTAATGACGTGTTCTTGCCTCAGCTCGATTCGGTAGGCTTGTTACAATTGGGCGGATCTAGA
PolII/polyA_*Xba* I rev	TCTAGATCCGCCCAATTGTAACAAGCCTACCGAATCGAGCTGAGGCAAGAACACGTCATTAGAGAGAAGCGAAGTAACTGAAGCCGAAGCTT
PolII/U1box_*Hin* dIII fw	AAGCTTGTAGGAGGATAGCGGTCGACCTCTCTAATGACGTGTTCTTGCCTCAGCTCGATTCGGTAGTAAAGCCTCGCAGTTCCGAACTTTCTGGAGTTTCAAAAGTAGACTCTAGA
PolII/U1box_*Xba* I rev	TCTAGAGTCTACTTTTGAAACTCCAGAAAGTTCGGAACTGCGAGGCTTTACTACCGAATCGAGCTGAGGCAAGAACACGTCATTAGAGAGGTCGACCGCTATCCTCCTACAAGCTT

RT-qPCR primes for expression constructs detection

Name	***Sequence 5' to 3'***
PolIII/U6term fw	TGCAATCGGATCGCACTTTT
PolIII/U6term rev	AAGGGACAATCTCTGCGGTG
PolII/polyA fw	CGGCTTCAGTTACTTCGCTT
PolII/polyA rev	TCCGCCCAATTGTAACAAGC
PolII/U1-3'box fw	GTAGGAGGATAGCGGTCGAC
PolII/U1-3'box rev	TCGGAACTGCGAGGCTTTA

DNA-oligonucleotides used for lenght constructs (LC) cloning (restrictions sites underlined)

Name	***Sequence 5' to 3'***
LC_{80}_*Hin* dIII fw	AAAGCTTTGCAATCGGATCGCACTTTTCTCTCTAATGACGTGTTCTTGCCTCAGCTCGATTCGGTAGCACCGCAGAGATTGTCCCTTTCTAGA
LC_{80}_*Xba* I rev	TCTAGAAAGGGACAATCTCTGCGGTGCTACCGAATCGAGCTGAGGCAAGAACACGTCATTAGAGAGAAAAGTGCGATCCGATTGCAAAGCTTT
LC_{120}_*Hin* dIII fw	AAGCTTCGGCTTCAGTTACTTCGCTTCTCTCTAATGACGTGTTCTTGCCTCAGCTCGATTCGGTAGACTGCCATCACTAGCCACCGCTTCCAATTCCCTACTCTGAGCTTGTTACAATTGGGCGGATCTAGA
LC_{120}_*Xba* I rev	TCTAGATCCGCCCAATTGTAACAAGCTCAGAGTAGGGAATTGGAAGCGGTGGCTAGTGATGGCAGTCTACCGAATCGAGCTGAGGCAAGAACACGTCATTAGAGAGAAGCGAAGTAACTGAAGCCGAAGCTT

LC_{200}_*Hin* dIII fw	AAGCTTGTAGGAGGATAGCGGTCGACCTCTCTAATGACGTGTTCTTGCCTCAGCTCGATTCGGTAGACTGCCATCACTAGCCACCGCTTCCAATTCCCTACTCTGACTCTCTAATGACGTGTTCTTGCCTCAGCTCGATTCGGTAGACTGCCATCACTAGCCACCGCTTCCAATTCCCTACTCTGATAA AGCCTCGCAGTTCCGATCTAGA
LC_{200}_*Xba* I rev	TCTAGATCGGAACTGCGAGGCTTTATCAGAGTAGGGAATTGGAAGCGGTGGCTAGTGATGGCAGTCTACCGAATCGAGCTGAGGCAAGAACACGTCATTAGAGAGTCAGAGTAGGGAATTGGAAGCGGTGGCTAGTGATGGCAGTCTACCGAATCGAGCTGAGGCAAGAACACGTCATTAGAGAGGTCG ACCGCTATCCTCCTACAAGCTT
LC_{360}_*Hin* dIII fw	AAGCTTGGATTAATGTCAGGCTCCGGCTCTCTAATGACGTGTTCTTGCCTCAGCTCGATTCGGTAGACTGCCATCACTAGCCACCGCTTCCAATTCCCTACTCTGACTCTCTAATGACGTGTTCTTGCCTCAGCTCGATTCGGTAGACTGCCATCACTAGCCACCGCTTCCAATTCCCTACTCTGACTC TCTAATGACGTGTTCTTGCCTCAGCTCGATTCGGTAGACTGCCATCACTAGCCACCGCTTCCAATTCCCTACTCTGACTCTCTAATGACGTGTTCTTGCCTCAGCTCGATTCGGTAGACTGCCATCACTAGCCACCGCTTCCAATTCCCTACTCTGACTGCGCGGTAGGGTAACTTATCTAGA
LC_{360}_*Xba* I rev	TCTAGATAAGTTACCCTACCGCGCAGTCAGAGTAGGGAATTGGAAGCGGTGGCTAGTGATGGCAGTCTACCGAATCGAGCTGAGGCAAGAACACGTCATTAGAGAGTCAGAGTAGGGAATTGGAAGCGGTGGCTAGTGATGGCAGTCTACCGAATCGAGCTGAGGCAAGAACACGTCATTAGAGAGTCA GAGTAGGGAATTGGAAGCGGTGGCTAGTGATGGCAGTCTACCGAATCGAGCTGAGGCAAGAACACGTCATTAGAGAGTCAGAGTAGGGAATTGGAAGCGGTGGCTAGTGATGGCAGTCTACCGAATCGAGCTGAGGCAAGAACACGTCATTAGAGAGCCGGAGCCTGACATTAATCCAAGCTT
LC_{680}_*Hin* dIII fw	AAGCTTCAGCCCTGGACACCTGATTACTCTCTAATGACGTGTTCTTGCCTCAGCTCGATTCGGTAGACTGCCATCACTAGCCACCGCTTCCAATTCCCTACTCTGACTCTCTAATGACGTGTTCTTGCCTCAGCTCGATTCGGTAGACTGCCATCACTAGCCACCGCTTCCAATTCCCTACTCTGACTC TCTAATGACGTGTTCTTGCCTCAGCTCGATTCGGTAGACTGCCATCACTAGCCACCGCTTCCAATTCCCTACTCTGACTCTCTAATGACGTGTTCTTGCCTCAGCTCGATTCGGTAGACTGCCATCACTAGCCACCGCTTCCAATTCCCTACTCTGACTCTCTAATGACGTGTTCTTGCCTCAGCTCGA TTCGGTAGACTGCCATCACTAGCCACCGCTTCCAATTCCCTACTCTGACTCTCTAATGACGTGTTCTTGCCTCAGCTCGATTCGGTAGACTGCCATCACTAGCCACCGCTTCCAATTCCCTACTCTGACTCTCTAATGACGTGTTCTTGCCTCAGCTCGATTCGGTAGACTGCCATCACTAGCCACCGC TTCCAATTCCCTACTCTGACTCTCTAATGACGTGTTCTTGCCTCAGCTCGATTCGGTAGACTGCCATCACTAGCCACCGCTTCCAATTCCCTACTCTGAACTGGCCAGAGTCTTGAGTCTCTAGA
LC_{680}_*Xba* I rev	TCTAGAGACTCAAGACTCTGGCCAGTTCAGAGTAGGGAATTGGAAGCGGTGGCTAGTGATGGCAGTCTACCGAATCGAGCTGAGGCAAGAACACGTCATTAGAGAGTCAGAGTAGGGAATTGGAAGCGGTGGCTAGTGATGGCAGTCTACCGAATCGAGCTGAGGCAAGAACACGTCATTAGAGAGTCA GAGTAGGGAATTGGAAGCGGTGGCTAGTGATGGCAGTCTACCGAATCGAGCTGAGGCAAGAACACGTCATTAGAGAGTCAGAGTAGGGAATTGGAAGCGGTGGCTAGTGATGGCAGTCTACCGAATCGAGCTGAGGCAAGAACACGTCATTAGAGAGTCAGAGTAGGGAATTGGAAGCGGTGGCTAGTG ATGGCAGTCTACCGAATCGAGCTGAGGCAAGAACACGTCATTAGAGAGTCAGAGTAGGGAATTGGAAGCGGTGGCTAGTGATGGCAGTCTACCGAATCGAGCTGAGGCAAGAACACGTCATTAGAGAGTCAGAGTAGGGAATTGGAAGCGGTGGCTAGTGATGGCAGTCTACCGAATCGAGCTGAGGCA AGAACACGTCATTAGAGAGTCAGAGTAGGGAATTGGAAGCGGTGGCTAGTGATGGCAGTCTACCGAATCGAGCTGAGGCAAGAACACGTCATTAGAGAGTAATCAGGTGTCCAGGGCTGAAGCTT

RT-qPCR primes for length constructs (LC) detection

Name	***Sequence 5' to 3'***
LC $_{80}$ fw	TGCAATCGGATCGCACTTTT
LC $_{80}$ rev	AAGGGACAATCTCTGCGGTG
LC $_{120}$ fw	CGGCTTCAGTTACTTCGCTT
LC $_{120}$ rev	TCCGCCCAATTGTAACAAGC
LC $_{200}$ fw	GTAGGAGGATAGCGGTCGAC
LC $_{200}$ rev	TCGGAACTGCGAGGCTTTA
LC $_{360}$ fw	GGATTAATGTCAGGCTCCGG
LC $_{360}$ rev	TAAGTTACCCTACCGCGCAG
LC $_{680}$ fw	CAGCCCTGGACACCTGATTA
LC $_{680}$ rev	GACTCAAGACTCTGGCCAGT

Northern blot probe GAPDH

Name	***Sequence 5' to 3'***
Probe 1 fw	ACAGTCAGCCGCATCTTCTT
Probe 1 rev	TAATACGACTCACTATAGGGTGTGGTCATGAGTCCTTCCA
Probe 2 fw	ACCCAGAAGACTGTGGATGG
Probe 2 rev	TAATACGACTCACTATAGGGAGGGGTCTACATGGCAACTG
